# When health data go dark: the importance of the DHS Program and imagining its future

**DOI:** 10.1186/s12916-025-04062-6

**Published:** 2025-04-24

**Authors:** Jessie Jane Khaki, Jil Molenaar, Sulata Karki, Emmanuel Olal, Manuela Straneo, Marie Alice Mosuse, Jovanny Tsuala Fouogue, Bernadette Hensen, Adama Baguiya, Angèle Musau Nkola, Kerry L. M. Wong, Oumar Aly Ba, Amani Kikula, Fassou Mathias Grovogui, Aline Semaan, Anteneh Asefa, Peter M. Macharia, Chido Dziva Chikwari, Mariame Oumar Ouédraogo, Aliki Christou, Emelda A. Okiro, Martin Kavao Mutua, Abioye Amodu, Mwelwa Phiri, Rukundo Athanase, David Lagoro Kitara, Onikepe Owolabi, Andrea B. Pembe, Bosede B. Afolabi, Lenka Beňová

**Affiliations:** 1 Kamuzu University of Health Sciences, Blantyre, Malawi; 2 Malawi Liverpool Wellcome Programme, Blantyre, Malawi; 3 Institute of Tropical Medicine Antwerp, Antwerp, Belgium; 4 University of Antwerp, Antwerp, Belgium; 5 HERD International, Lalitpur, Nepal; 6 Adroit Consult International, Kampala, Uganda; 7 Karolinska Institutet, Stockholm, Sweden; 8 Vrije Universiteit Brussel, Brussel, Belgium; 9 Department of Gynaecology, Obstetrics and maternal health, University of Dschang, Dschang, Cameroon; 10 Institut de Recherche en Sciences de la Santé (IRSS), Ouagadougou, Burkina Faso; 11 Ecole de Sante Publique, University of Lubumbashi, Lubumbashi, Democratic Republic of the Congo; 12 Independent researcher, London, UK; 13 GeoHealth group, Institute of Global Health, Faculty of Medicine, University of Geneva, Geneva, Switzerland; 14 Institute for Environmental Sciences, University of Geneva, Geneva, Switzerland; 15 Muhimbili University of Health and Allied Sciences, Dar es Salaam, Tanzania; 16 African Centre of Excellence for the Prevention and Control of Communicable Diseases (CEA-PCMT), Gamal Abdel Nasser University, Conakry, Republic of Guinea; 17 Athena Institute, Vrije Universiteit, Amsterdam, The Netherlands; 18 Population & Health Impact Surveillance Group, Kenya Medical Research Institute-Wellcome Trust Research Programme, Nairobi, Kenya; 19 Biomedical Research and Training Institute, Harare, Zimbabwe; 20 London School of Hygiene and Tropical Medicine, London, UK; 21 Dalla Lana School of Public Health, University of Toronto, Toronto, Canada; 22 African Population and Health Research Centre, Dakar, Senegal; 23 Independent researcher, Lagos, Nigeria; 24 Zambart, Lusaka, Zambia; 25 Ministry of Health, Kigali, Rwanda; 26 Gulu University, Faculty of Medicine, Gulu, Uganda; 27 Guttmacher Institute, New York, USA; 28 Faculty of Clinical Sciences, College of Medicine, University of Lagos, Lagos, Nigeria; 29 Department of Obstetrics and Gynaecology, Lagos University Teaching Hospital, Lagos, Nigeria; 30 Centre for Clinical Trials and Implementation Science (CCTRIS), College of Medicine, University of Lagos, Lagos, Nigeria

**Keywords:** Population surveys, Low- and middle-income countries, Demographic and Health Survey, Service Provision Assessment, DHS Program, USAID, Funding, Health data, Demographic data

## Abstract

**Background:**

The suspension and/or termination of many programmes funded through the United States Agency for International Development (USAID) by the new US administration has severe short- and long-term negative impacts on the health of people worldwide. We draw attention to the termination of the Demographic and Health Surveys (DHS) Program, which includes nationally representative surveys of households, DHS, Malaria Indicator Surveys [MIS]) and health facilities (Service Provision Assessments [SPA]) in over 90 low- and middle-income countries. USAID co-funding and provision of technical support for these surveys has been shut down.

**Main body:**

The impact of these disruptions will reverberate across local, regional, national, and global levels and severely impact the ability to understand the levels and changes in population health outcomes and behaviours. We highlight three key impacts on (1) ongoing data collection and data processing activities; (2) future data collection and consequent lack of population-level health indicators; and (3) access to existing data and lack of support for its use.

**Conclusions:**

We call for immediate action on multiple fronts. In the short term, universal access to existing data and survey materials should be restored, and surveys which were planned or in progress should be completed. In the long term, this crisis should serve as a tipping point for transforming these vital surveys. We call on national governments, regional organisations, and international partners to develop sustainable alternatives that preserve the principles (standardised questionnaires, backward compatibility, open access data with rigorous documentation) which made the DHS Program an invaluable global health resource.

## Background

**Fig. 1:**
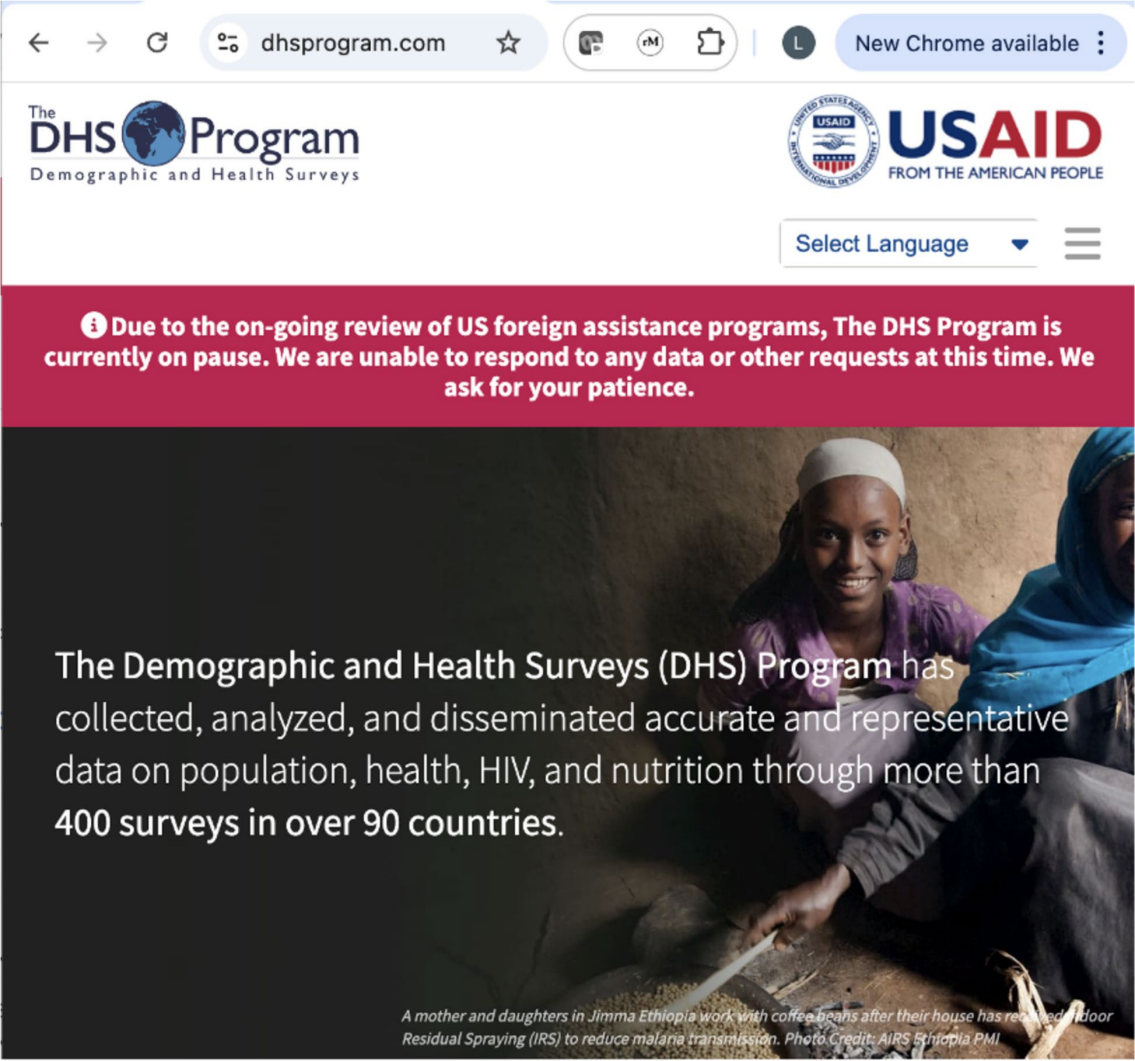
Screenshot of disruption in data access on DHS Program website, February 2, 2025 (source: authors)

The suspension and/or termination of many programmes funded through the United States Agency for International Development (USAID) has severe short- and long-term negative impacts on the health of people worldwide [1, 2]. USAID funding cuts have also extended to the co-financing and provision of technical support to the DHS Program [3] which includes nationally representative surveys of households (Demographic and Health Surveys [DHS], Malaria Indicator Surveys [MIS]) and health facilities (Service Provision Assessments [SPA]) in over 90 low- and middle-income countries (LMIC). These surveys capture high-quality data to track health-related Sustainable Development Goals (SDGs) indicators and beyond [4]. For many LMICs, surveys implemented through the DHS Program represent the most comprehensive, continuous, and reliable source of health data. The sudden termination in USAID funding means that despite agreements in place with dozens of LMICs, DHS activities have ground to a halt. While a small subset of USAID programs remains active as of late March 2025, including limited funding for some in-country DHS work, the future of these initiatives is uncertain [5]. One immediate consequence is that since late January 2025, US-based DHS Program staff cannot approve new users or requests for access to DHS data (Fig. 1). The Data Rescue Project has successfully preserved population-level estimates and spatial data, but this cannot replace the comprehensive microdata and customisable analyses previously available through the DHS Program platform [6].

In this article, we draw attention to the consequences of the DHS Program’s suspension on our ability to track population health outcomes and behaviours. We aim to raise awareness about what this means for national decision-makers and the global health community, and discuss potential paths forward. We examine the impact of these disruptions at three levels and call on national governments, regional organisations, and international partners to develop sustainable alternatives for continued collection and use of these essential survey data.

## The use of DHS Program data in policy making

Since 1984, 452 surveys have been conducted in over 90 LMICs as part of the DHS Program, and their findings have served as a vital source for country stakeholders, enabling health system monitoring and planning (Table 1). Making data freely accessible has been a core principle of the DHS Program since its inception, with all datasets available at no cost to users worldwide. To date, over 300 reports and more than 6000 peer-reviewed papers have been published based on DHS data [7].

**Table 1:** Examples of policy impact of data collected by the DHS Program

**Tanzania**: The fifth Health Sector Strategic Plan (HSSP V) explicitly states that “household surveys are the most important source to track national progress in health status” [8]. DHS indicators—including institutional birth rate, modern contraceptive use, and malaria parasite prevalence in children—directly inform health planning. High chronic malnutrition rates documented in the 2015/2016 DHS underpinned the country’s first National Multisectoral Nutrition Action Plan [9].
**Nepal**: The elevated maternal mortality data revealed in the 2016 DHS prompted the government to enhance maternal health services by establishing birthing centers with skilled birth attendants trained at local health facilities [10].
**Guinea**: DHS data enabled subnational tailoring of malaria interventions, supporting evidence-based prioritisation for the recently rolled out malaria vaccine and more effective resource allocation in endemic regions [11].
**Pakistan**: Childhood mortality data from the 2017–2018 DHS directly informed the development of a new support program for pregnant women, demonstrating how survey findings translate into targeted interventions [12].
**Global**: DHS data are used to calculate and track targets related to SDG 5.6, which has been instrumental in advancing gender equality and reproductive agency in many countries [13]. For example, several Nigerian states have strengthened laws against child marriage, influenced by SDG 5.6 and the African Union’s campaign against early marriage [14, 15].

## Impact of disruptions

The impact of disruptions to the DHS Program will reverberate across local, subnational, national, regional and global levels. We highlight three such impacts here, starting with the most immediate.

### Disruptions to ongoing activities

First, disruptions to ongoing activities mean that data planned for collection or already collected will not be analysed or made available. A comprehensive overview of all affected surveys is provided in Table 2 [16]. We highlight several significant disruptions below:

- In Malawi, Zimbabwe, Zambia and Nigeria, DHS data have been collected, but full reports and datasets have not yet been released. In Malawi, current DHS data guide PEPFAR-funded antiretroviral therapy programs for roughly 1 million HIV+ patients [17]. Nigeria is the largest country in Africa and has among the highest burdens of preventable maternal and perinatal mortality in the world [18]. In 2024, Nigeria DHS was expanded to capture maternal deaths and conduct verbal autopsies, but these crucial data might now never be available to decision-makers to target the causes and determinants of these preventable deaths.
- The complete data for the Democratic Republic of the Congo (DRC) DHS survey from 2023 to 2024 are not yet available. However, a recently published Key Findings report (August 2024) indicates that data collection is complete [19]. The last nation-wide data from the DRC dates from 2013 to 2014, more than 10 years ago. As one of Africa’s largest countries, with vast geographic challenges, ongoing armed conflict, and a fragile health system, the country faces significant obstacles in collecting alternative data for guiding policies.
- The Uganda MIS and Ethiopia DHS were collecting data; activities will have been interrupted or significantly affected.
- Significant uncertainties surround the 2025 and 2026 surveys planned in multiple countries—including DHS in the Philippines and Togo, and SPA in Nepal—jeopardizing data collection and the livelihoods of field staff.

**Table 2:**
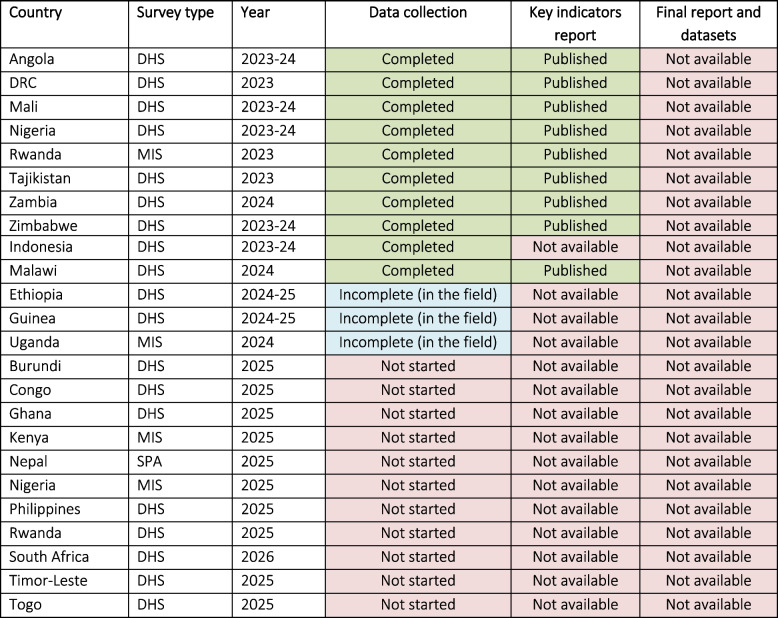
Overview of affected DHS surveys (status 31 March 2025 based on the DHS Program website)

### Impact on monitoring population health indicators

Second, without future data collection, monitoring of population health indicators will be severely compromised.

- The suspension of new surveys deprives LMICs of vital population-level data needed for evidence-based policymaking. For example, without key indicators on HIV prevalence and testing uptake, persisting gaps and inequalities in HIV services will not be efficiently addressed. Similarly, a lack of comprehensive human papillomavirus (HPV) vaccine coverage data will mean countries cannot monitor the rollout of this crucial intervention. The DHS provides nationally representative coverage data on HPV vaccine uptake that other current systems, such as the District Health Information System 2 (DHIS2), do not fully capture. Without such data, countries remain unable to assess who has been reached and adjust their programmes accordingly.
- The absence of accessible, high-quality data undermines transparency, accountability, and evidence-based decision-making in healthcare. Data must be openly available for governments, researchers, and civil society organisations to work together effectively to advance health equity.
- The historical continuity of population health data is threatened. This data gap would prevent identification of critical trends in fertility, mortality, health outcomes and health behaviours—both within countries (across regions and urban/rural divides) and between countries. Without such data, we cannot effectively analyze how major forces like climate change, urbanisation, and healthcare access affect health. Ironically, the discontinuation of DHS surveys also significantly hinders our ability to quantify the impact of USAID funding reductions themselves on population health outcomes—a critical “natural experiment” that could inform future foreign aid policy and demonstrate the value of sustained international health investments.

### Loss of trust in research and training opportunities for future scientists

Third, the disruption to access to existing data and lack of support for its use will have devastating consequences on populations’ trust in research and on training of scientists.

- Survey participants—predominantly women—shared their personal information with the understanding it would inform research and policy decisions. DHS Program data represent a vital community resource and have been called a national and international public good [20]. Restricting access to these data violates the ethical principles and commitments made to communities during data collection.
- While each country’s Ministry of Health and/or national statistics agency have the datasets collected in the past, storage and access to such data and related documentation is not assured or practically arranged.
- Without a centralised data portal, researchers lose the ability to conduct comparisons of vital indicators across time and countries. This includes the StatCompiler dashboard, a critical resource for non-technical users of DHS findings, and support for data users. It will prevent policymakers and other stakeholders from tracking progress towards international targets, such as the SDGs. Additionally, in the absence of this high-quality harmonised and backward-compatible repository, evaluating the effectiveness of interventions and programmes involving multiple countries will be compromised.
- The termination of funding will impact training of health professionals, researchers, and scientists in two ways: (1) through suspension of training courses on survey methodologies; and (2) through lack of access to survey data which are commonly used by undergraduate, master’s and PhD students for their theses, especially in LMICs. This significantly reduces the preparation of future national and global health leadership.

## Conclusions

We are a group of health workers, national policymakers, students, educators, and researchers who have extensively relied on the data, methods, and findings produced by the DHS Program and used these data for decision-making. We call for restoration of universal access to existing data. In the short term, all the survey-related activities which were already in progress should be completed. Interrupting ongoing processes is a substantial waste of resources from multiple donors and risks losing critical technical expertise. In addition, it violates the social and moral contract stemming from collecting data from individuals with the purpose of improving their families’ and communities’ health and well-being. In the long term, this crisis necessitates transforming how vital population surveys are conducted and funded. With signals that USAID funding is unlikely to be fully reinstated, we must urgently explore alternative platforms that retain the DHS Program’s most valuable characteristics: cross-country comparability, open access to data, and shared technical expertise. Standardisation of survey methodologies, sampling techniques, questionnaires, and data coding is crucial for enabling cross-country and temporal comparisons—a feature that becomes compromised when countries undertake these surveys in isolation. A centralised data repository with sustainable provisions for data storage, technical assistance, and accessibility, protected from political influence and funding volatility, remains essential. We recommend establishing such a repository within LMICs and distributing it across servers in multiple locations and organisations to prevent dependence on any single organisation or funder, while building capacity where the data originates.

This moment demands reimagining a system less dependent on external funding. By further strengthening technical capacity in LMICs and reducing financial dependence of national ministries of health, statistics bureaus, and other relevant bodies that already lead many DHS activities, we can build more sustainable, self-reliant health monitoring systems. The alternative—a widespread reduction in the frequency and quality of population health surveys—would create dangerous blind spots in our understanding of demographic and health trends, particularly in countries where surveys conducted by the DHS Program have been the main source of population health data, and therefore crucial for effective health planning and response. The cost of these data going dark, measured in our inability to identify and effectively respond to health crises and population needs, far exceeds the investment required to maintain these surveys. We call on global health leaders, regional organisations, and international partners to develop sustainable alternatives that preserve the scientific rigor and public accessibility that made the DHS Program an invaluable global health resource. Only through collective commitment can we bring these vital health insights back to light and ensure the health of all the world’s populations remains visible.

## Data Availability

No datasets were generated or analysed during the current study.

## Abbreviations

USAID: United States Agency for International Development
DHS: Demographic and Health Survey
MIS: Malaria Indicator Survey
SPA: Service Provision Assessment
LMICs: Low- and middle-income countries
SDGs: Sustainable Development Goals
HSSP: Health Sector Strategic Plan (Tanzania)
PEPFAR: U.S. President’s Emergency Plan for AIDS Relief
HPV: Human papillomavirus
DHIS2: District Health Information System 2
